# Sperm navigation along helical paths in 3D chemoattractant landscapes

**DOI:** 10.1038/ncomms8985

**Published:** 2015-08-17

**Authors:** Jan F. Jikeli, Luis Alvarez, Benjamin M. Friedrich, Laurence G. Wilson, René Pascal, Remy Colin, Magdalena Pichlo, Andreas Rennhack, Christoph Brenker, U. Benjamin Kaupp

**Affiliations:** 1 Molecular Sensory Systems, Center of Advanced European Studies and Research (caesar), Ludwig-Erhard-Allee 2, 53175 Bonn, Germany; 2 Biological Physics, Max Planck Institute for the Physics of Complex Systems, Nöthnitzer Straße 38, 01187 Dresden, Germany; 3 Department of Physics, University of York, YO10 5DD Heslington, York, UK; 4 Max Planck Institute for Terrestrial Microbiology, Karl-von-Frisch Straße 16, 35043 Marburg, Germany; 5 Centre of Reproductive Medicine and Andrology, University of Muenster, 48149 Muenster, Germany

## Abstract

Sperm require a sense of direction to locate the egg for fertilization. They follow gradients of chemical and physical cues provided by the egg or the oviduct. However, the principles underlying three-dimensional (3D) navigation in chemical landscapes are unknown. Here using holographic microscopy and optochemical techniques, we track sea urchin sperm navigating in 3D chemoattractant gradients. Sperm sense gradients on two timescales, which produces two different steering responses. A periodic component, resulting from the helical swimming, gradually aligns the helix towards the gradient. When incremental path corrections fail and sperm get off course, a sharp turning manoeuvre puts sperm back on track. Turning results from an ‘off' Ca^2+^ response signifying a chemoattractant stimulation decrease and, thereby, a drop in cyclic GMP concentration and membrane voltage. These findings highlight the computational sophistication by which sperm sample gradients for deterministic klinotaxis. We provide a conceptual and technical framework for studying microswimmers in 3D chemical landscapes.

## Abstract (web summary)

Sperm use external cues to find the egg using ill-defined principles. Here the authors use holographic microscopy and optochemical tools to study sperm swimming in light-sculpted chemical 3D landscapes; they show that sperm translate the temporal stimulation pattern into multiple swimming behaviours to orient deterministically in a gradient.

## Graphical abstract

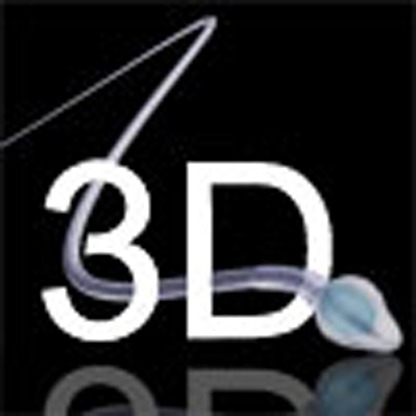

Many motile sperm rely on chemical and physical cues to locate the egg^1^,^2^,^3^,^4^. A beating hair-like filament, called the flagellum, serves both as an antenna that gathers sensory cues and as a motor that propels the cell. Receptors on the flagellar surface transduce these sensory cues into cellular signals. Ultimately, these signals modulate the wave-like beating of the flagellum that steers a sperm's swimming path (for review see ref. 5).

Sperm from many species, in particular marine animals, are attracted to the egg by chemical factors—a process called chemotaxis. Previous chemotaxis studies tracked sperm only in two-dimensions (2D); at the glass/water interface of shallow observation chambers, sperm swim on a plane in circles^6^,^7^,^8^,^9^,^10^. While cruising on circular paths in a chemical gradient, sperm sample the chemoattractant concentration either continuously or intermittently and gradually adjust their swimming path—a mechanism called klinotaxis. The repetitive stimulation entrains a suite of Ca^2+^ bursts that modulate the waveform of the flagellar beat^5^,^9^,^11^,^12^. The ensuing alternate periods of symmetrical and asymmetrical beating give rise to a looping swimming pattern (‘drifting circles') up a gradient. However, unrestricted sperm from species such as sea urchin swim on a 3D helical path^13^,^14^,^15^,^16^,^17^. The conceptual work by Crenshaw^17^,^18^ suggests that in a chemical gradient, sperm orient by helical klinotaxis, that is, by alignment of the helix towards the gradient. In addition, Crenshaw^17^,^18^ shows that such alignment could be theoretically achieved if the components of the cell's rotational velocity are simple functions of the stimulus.

A generic quantitative theory of this chemotactic steering by Friedrich and Jülicher^19^ captures the essence of navigation along periodic paths: a cellular signalling system transforms the periodic stimulation *s*(*t*) (chemoattractant binding) into a periodic intracellular signal *i*(*t*) (Ca^2+^ bursts) that in turn modulates the swimming path curvature *κ*_p_(*t*). The phase lag between *s*(*t*) and *κ*_p_(*t*), that is, the latency of the Ca^2+^ signal, is a crucial determinant of the directed drift of circles up or down a 2D chemical gradient. This theory also predicts bending of the swimming helix in 3D. However, these theories have not been experimentally tested in a well-defined 3D chemical gradient; moreover, the mechanisms of 3D klinotaxis cannot be derived from 2D swimming at interfaces, not least because of the hydrodynamic interactions between the flagellum and the surface of the recording chamber^20^,^21^. In fact, 3D navigation mechanisms remain unknown for any swimming eukaryotic cell, because of the technical challenges to rapidly establish and quantitatively characterize a chemical gradient and to track rapidly moving cells as they traverse a complex chemical 3D landscape.

We chose the sea urchin *Arbacia punctulata* to study the search strategy of sperm in a 3D chemoattractant landscape. *A. punctulata* are broadcast spawners that release their gametes into the ocean, where sperm swim freely. For several reasons, *A. punctulata* sperm provide an unmatched model to address fundamental questions of cell navigation^22^: (1) chemotaxis has been well-established^22^,^23^; (2) the chemoattractant is known^24^, and the signalling pathway has been studied in depth^25^,^26^,^27^,^28^,^29^; (3) conditions for swimming in an aqueous medium can be readily emulated; finally, (4) *Arbacia* sperm, unlike mammalian sperm, represent a homogenous population, that is, most sperm are chemotactically active.

Here we study freely swimming sperm using digital inline high-speed holographic microscopy^30^. Moreover, 3D landscapes with defined spatio-temporal pattern are created by light using a caged chemoattractant^31^. In addition, we study by reverse optochemical engineering of signalling events, how steering responses are adjusted during navigation in a gradient. Finally, using hydrodynamic simulations and a minimal model of sperm signalling, we provide a theoretical framework accounting for sperm navigation in 3D.

## Results

### Tracking sperm in 3D

**Figure 1:**
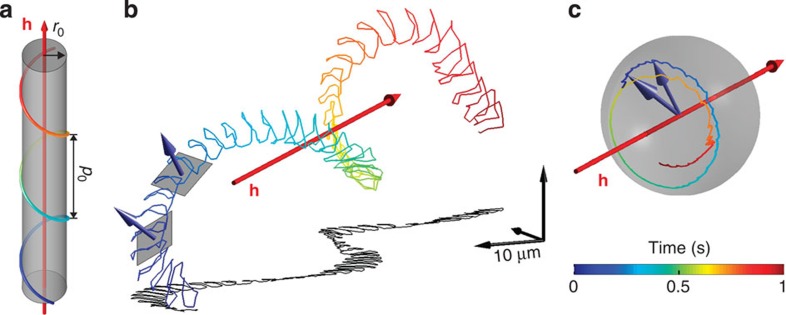
Sperm navigate along helical paths. (**a**) Diagram showing the averaged swimming path of unstimulated sperm (duration 1 s, see color bar). Radius *r*_0_ and pitch *p*_0_ are drawn to scale. (**b**) Reconstruction of the 3D swimming path far from walls. Head wiggling was used to determine the beating plane orientation. (**c**) The vector normal to the beating plane (blue arrows) precesses around the helical axis (**h**, red arrow) with fixed inclination, describing a circle on the surface of a unit sphere centred around **h**. Vectors are not to scale.

Sperm were illuminated with coherent laser light. Light scattered by sperm interfered with the unscattered background, and this interference was recorded by a camera at 600 holograms per s (h.p.s.). Applying the Rayleigh–Sommerfeld back-propagator^32^, we determined numerically from each hologram the 3D coordinates of the sperm head^33^ (Supplementary Fig. 1). Far from boundaries, *A. punctulata* sperm swam on a regular helical path with a mean speed *v*=200±57 μm s^−1^ (Fig. 1a); mean helix parameters were: radius *r*_0_=8.4±3.1 μm, pitch *p*_0_=47.6±9.1 μm, helix period *T*=0.38±0.07 s, path curvature *κ*_p_=0.065±0.013 μm^−1^ and path torsion *τ*_p_=0.067±0.031 μm^−1^ (track duration 1 s; *n*=20 cells, mean±s.d.). For comparison, near a glass/water interface, sperm swim on circles of radius *r*_0_=23.5±0.9 μm with swimming speed *v*=160±29 μm s^−1^, and circle period *T*=0.9±0.2 s (*n*=6). Thus, 2D and 3D kinematic parameters are distinctively different, underscoring the notion that 2D and 3D navigation are fundamentally different^20^.

The sperm head wiggles around the average path with a frequency identical to that of the flagellar beat^34^; passive head wiggling counterbalances periodic forces generated by active flagellar bending^35^. For freely swimming sperm, head wiggling indicated a flagellar beat frequency of 43.5±3.5 Hz (Fig. 1b; *n*=20). Moreover, head wiggling was approximately planar, consistent with an approximately planar beat pattern. While sperm swim along a helix, the head-wiggling plane slowly rotates around the helix axis: the normal vector describes a circle on the unit sphere (Fig. 1c), characterized by a constant inclination to the helix axis (52.0°±11.5°; circular mean±circular s.d., *n*=20).

**Figure 2:**
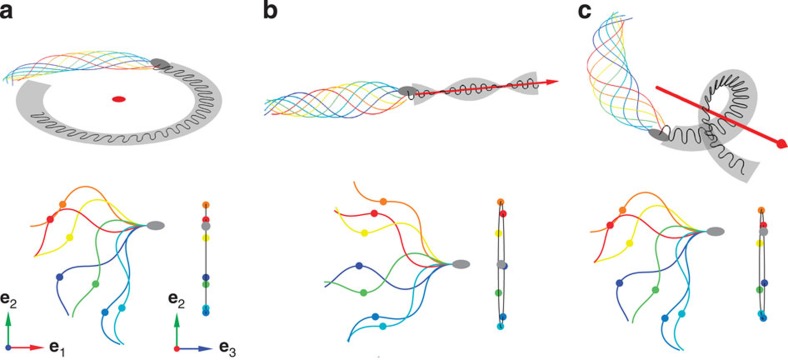
Simulated swimming paths for three prototypical flagellar waveforms. The top of each panel illustrates the swimming path, while at the bottom is shown a waveform sequence for aligned sperm head (grey) with a coloured reference point on the flagellum (left: top view; right: side view). (**a**) Planar and asymmetrical beating (mean flagellar curvature, *K*_0_>0) results in circular paths. (**b**,**c**) A small flagellar twist (*τ*_f_>0) results in non-planar beat patterns and swimming paths. For symmetric beating (*K*_0_=0), the resulting swimming path is a twisted ribbon (**b**), whereas for asymmetric beating (*K*_0_>0) sperm swim on helices, and the beating plane has a constant inclination with the helix vector **h** (**c**).

To gain insight into the mechanisms producing a helical path, we simulated swimming paths resulting from different flagellar waveforms using resistive-force theory^35^,^36^. The flagellar shape was characterized by a constant flagellar twist *τ*_f_ and a flagellar curvature *κ*_f_(*l*,*t*)=*K*_0_+*B* cos(*ω*_0_*t*−*λl*) describing a travelling bending wave with beat amplitude *B*, wave speed *ω*_0_/*λ* and mean flagellar curvature *K*_0_ along the arc length *l* of the flagellum. We calculated the swimming path produced by three different flagellar waveforms: asymmetric/non-twisted (*K*_0_>0; *τ*_f_ =0), symmetric/twisted (*K*_0_=0; *τ*_f_ >0) and asymmetric/twisted (*K*_0_>0; *τ*_f_ >0). Without twist, the beat pattern and swimming path were planar (Fig. 2a), that is, sperm moved on circles of radius *r*_p_≈1.3/*K*_0_ that is set by the flagellar beat asymmetry. The symmetric/twisted beat produced a non-planar beat pattern, and sperm swam on twisted ribbons (Fig. 2b). A fraction of human sperm adopts this pattern^37^. Finally, an asymmetric/twisted beat produced a helical path, which is observed for some sperm species^13^,^14^, and head wiggling occurred in a plane that slowly rotated around the helix axis (Fig. 2c). Both features are borne out by our experiments (Fig. 1b). This beat pattern could result from a combination of periodic motor activity travelling down the flagellum, which drives planar bending waves and persistent motor activity, which twists the flagellum^38^.

### 3D steering is deterministic

**Figure 3:**
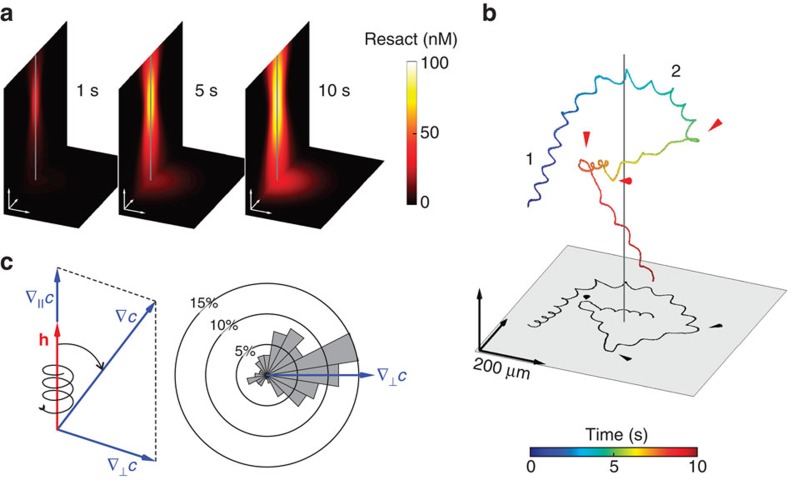
Tracking sperm in 3D chemoattractant gradients. (**a**) 3D resact gradients were established by photolysis of caged resact with a Gaussian ultraviolet-beam. The calculated free resact concentration is shown as a function of space and time, accounting for continuous photo-release and diffusion. The concentration field is rotationally symmetric about an axis shown by the vertical grey line. (**b**) Sperm chemotaxis in a 3D resact gradient. A grey line indicates the centre of the photolyzing beam. Initially, sperm swim on a perfect helix (1). While approaching the resact field, the helix axis bends smoothly towards the gradient centre (2). When small gradual corrections of the swimming path fail and sperm get off course, a sharp directional turn is initiated (arrowheads). (**c**) Left: the gradient ∇*c* (blue) can be decomposed into components parallel ∇_||_*c* and perpendicular ∇_⊥_*c* to the helical axis (**h**; red). The helical axis aligns with the gradient when it rotates towards ∇_⊥_*c*. Right: the histogram (*n*=10 cells) shows that the direction into which the helix axis changes scatters around ∇_⊥_*c* in a deterministic rather than random manner.

Using a photosensitive caged form of the chemoattractant resact, we sculpted by light well-defined 3D profiles of chemoattractant. The intensity profile of the uncaging ultraviolet light was approximated by a Gaussian beam (Supplementary Fig. 2, Supplementary Movie 1). To mimic an egg that continuously releases resact, ultraviolet illumination was maintained during the measurement. The time-dependent concentration profile *c*(**x**,*t*) was reconstructed numerically from the light profile using the resact diffusion coefficient^39^ and the extinction coefficient and quantum yield of caged resact (Fig. 3a, Supplementary Movie 2). From the swimming path **r**(*t*) and the concentration profile *c*(**x**,*t*), we derived the chemoattractant stimulus encountered by sperm *s*(*t*)=*c*(**r**(*t*),*t*) (Fig. 3b, Supplementary Fig. 3, Supplementary Movies 3,4).

Sperm might employ either a deterministic or stochastic strategy of chemotactic steering. For deterministic steering, correcting path adjustments are directed towards the gradient. Alternatively, during stochastic steering, path adjustments are chosen at random; afterwards, the microswimmer decides whether this choice was favourable and acts accordingly. Bacteria follow a stochastic search strategy; they alternate between episodes of straight swimming (‘runs') and stochastic tumbling, which randomizes cell orientation and, thereby, produces a random walk^40^. However, ‘runs' up a gradient are longer, producing a biased drift towards the chemoattractant source. To distinguish between deterministic and stochastic search strategies, we analysed the distribution of helical adjustments relative to the local concentration gradient for sperm crossing a chemoattractant field. The bending of the helix axis (d**h**/d*t*) is biased towards the perpendicular component ∇_⊥_*c* of the local gradient, that is, the optimal direction for local alignment with the gradient (Fig. 3c). Thus, steering responses of sperm are deterministic rather than stochastic.

### The sensori-motor transfer function

**Figure 4:**
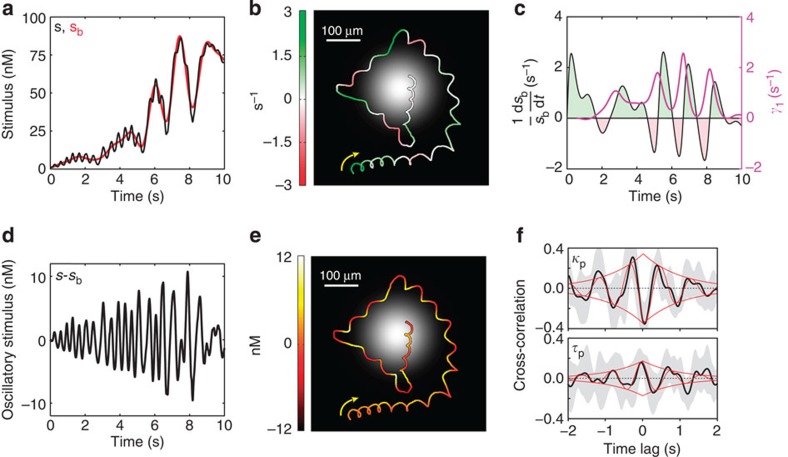
Chemical stimulus and cellular steering responses are coupled. (**a**) Attractant stimulus encountered by the sperm cell moving along the trajectory shown in Fig. 3b. The stimulus (black) can be decomposed into a slowly changing stimulus baseline (*s*_b_, red) and 2 Hz oscillations superimposed onto the baseline. (**b**) Top view of the swimming path. The light profile of the photolyzing beam at the focal plane is shown in grey shades. The time derivative of the stimulus baseline (d*s*_b_/d*t*) relative to the stimulus baseline is colour coded along the path. Shortly after down the gradient swimming (red), the cell turns abruptly (‘off response'). After each ‘off response', the cell swims up the gradient again (green). (**c**) Relative time derivative of *s*_b_ (magenta) and alignment rate (*γ*_1_; see Methods) of the helix axis with the concentration gradient (black). Phases of up the gradient and down the gradient swimming are characterized by increases (green) and decreases (red) of *s*_b_. The helix predominantly aligns with the gradient (*γ*_1_>0). However, sharp steering responses, characterized by high *γ*_1_, are observed whenever *s*_b_ decreases. (**d**) High-frequency component of the stimulus (approximately 2 Hz oscillations). (**e**) The oscillatory stimulus at *ca.* 2 Hz (colour coded) results from the periodic component of helical swimming in the gradient. (**f**) Cross-correlations between the high-frequency stimulus and modulations of path curvature (*κ*_p_; top) or torsion (*τ*_p_; bottom) with mean values shown in black and s.d. in grey (*n*=10 cells). Thin red lines show the fitted model of phase-locked oscillations and its exponential amplitude decay.

We identified two different steering modes while sperm navigate in a chemoattractant gradient. Steady bending of the helix aligned its axis with the gradient direction, thereby ramping up the mean stimulation level (‘on response'); occasionally bending was interrupted by a vigorous steering event that abruptly changed the swimming direction (‘off response') (Fig. 3b). Using a Fourier filter, we decomposed the stimulus function *s*(*t*) into high and low frequency components. One component represents stimulus oscillations of about 2 Hz, resulting from the helical nature of the path; this 2 Hz component was superimposed on the steady increase or decrease of the mean stimulus level (Fig. 4a,d). Remarkably, oscillations in curvature and torsion of the swimming helix are highly correlated with the stimulus oscillations, suggesting that subtle changes in helix geometry are underlying its smooth alignment with the gradient, that is, the ‘on response' (Fig. 4d,e).

We used a simple model of phase-locked oscillations to fit the cross-correlation of stimulus and swimming response: Acos(Ω_0_Δ*t*+*φ*)e^−*D*Δ*t*^, where Ω_0_ is the helix frequency, *φ* is the phase shift between path curvature or torsion and the stimulus, and *D* accounts for phase diffusion (Fig. 4f). We find a phase shift between stimulus oscillations and oscillations of curvature and torsion of *φ*_*κ*_=205.7° (184.8°, 233.2°) and *φ*_*τ*_=−16.3° (−72.2°, 43.6°), respectively, where the numbers in parentheses indicate 95% confidence intervals by bootstrap (*n*=10 cells). For optimal alignment, theory predicts^19^ a *φ*_*κ*_=180° and *φ*_*τ*_=0°—in fair agreement with our experimental results (see Supplementary Note 1).

Strong steering responses were initiated when the slow stimulus component began to decline, that is, when sperm were about to lose track of the gradient, hence ‘off response' (Fig. 4b,c). Nine out of 10 cells displayed such ‘off responses', and during a total recording time of 77 s ‘off responses' were observed on 31 occasions. Strong ‘off responses' provide sperm with the means to evoke an emergency steering manoeuvre when steady helix alignment (‘on response') is not sufficient.

In conclusion, sperm survey a chemical landscape on two different timescales simultaneously. While swimming up the gradient (mean stimulation level increases), the rapid periodic stimulus component smoothly and continuously bends the swimming helix towards the gradient. When swimming down the gradient (mean stimulation level decreases), sperm respond with a vigorous, yet almost optimally directed turn. While the smooth helix bending during the ‘on response' involves only subtle adjustments of helix parameters, the strong ‘off response' affords major transient distortions of helix geometry. After getting back on track, sperm resume regular helical swimming.

### The behavioural ‘off response' is generated by Ca^2+^

**Figure 5:**
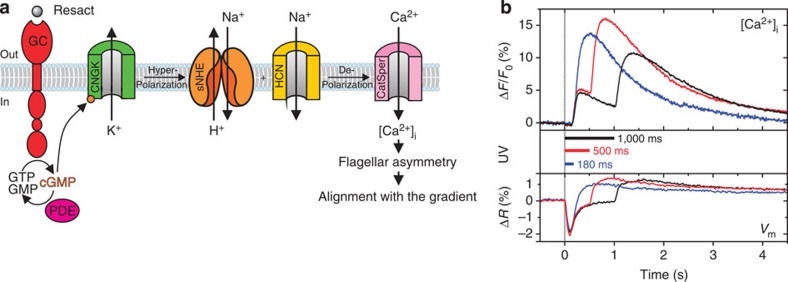
Signalling events underlying the ‘off responses'. (**a**) Signalling pathway underlying chemotaxis of *A. punctulata* sperm. The balance of synthesis by the chemoreceptor guanylyl cyclase (GC) and hydrolysis by PDE determines intracellular cGMP concentration. cGMP levels set the membrane potential of the cell via a cyclic nucleotide-gated K^+^-selective channel (CNGK). When sperm hyperpolarize, intracellular alkalization via a Na^+^/H^+^ exchanger (sNHE) and depolarization via hyperpolarization-activated and cyclic nucleotide-gated (HCN) channels prime the sperm-specific Ca^2+^ channel (CatSper) to open. When cGMP synthesis ceases, the cell quickly depolarizes, more CatSper channels open and a strong steering ‘off response' takes place. (**b**) Relative changes in [Ca^2+^]_i_ (Δ*F*/*F*_0_; top) and relative changes in membrane potential *V*_m_ detected with a ratiometric dye (Δ*R*; bottom) of sperm suspensions on release cGMP by light pulses of 180, 500 or 1,000 ms duration. Release of cGMP results in a rapid hyperpolarization and depolarization of sperm followed by a rise in [Ca^2+^]_i_ (‘on response'). When photolysis ceases, a second Ca^2+^ signal takes place (‘off response').

Sperm host a sophisticated pathway for Ca^2+^ signalling that controls the flagellar beat waveform (Fig. 5a). Briefly, chemoattractant stimulation produces a pulse of cyclic GMP (cGMP), the intracellular messenger^31^. The rise is caused by cGMP synthesis via the chemoreceptor, a guanylyl cyclase^27^ and the decay by cGMP hydrolysis via phosphodiesterase (PDE) activity. cGMP opens cyclic nucleotide-gated K^+^-selective channels^28^ (CNGK) and, thereby hyperpolarizes sperm^25^. During the decline from hyperpolarization, voltage-dependent Ca^2+^ channels (CatSper) open^26^. Ultimately, Ca^2+^ entering the sperm flagellum initiates a change in the flagellar waveform and a steering response^7^,^9^,^11^,^31^.

The behavioural ‘off response' when swimming down the gradient indicates that not only an increase, but also a decrease of chemoattractant evokes a Ca^2+^ signal. To test this idea, we developed a technique for optochemical control of a cellular response. We imposed a temporal pattern of photo-stimulation on sperm loaded with membrane-permeable caged cGMP^31^,^39^. The cGMP dynamics inside sperm is controlled via the balance between extrinsic ‘synthesis' by photolysis of caged cGMP and intrinsic hydrolysis by PDE. Thereby, we mimicked an increase or decrease of resact. We produced cGMP with 393-nm light and simultaneously followed the changes in Ca^2+^ concentration and membrane potential *V*_m_ by a Ca^2+^ indicator dye (Fluo-4) and a voltage probe (di-8-ANEPPS), respectively. As a control, we mimicked a brief puff of chemoattractant by a light pulse that is shorter than the latency of the Ca^2+^ response (<200 ms). The control stimulus, after a short latency, evoked a transient Ca^2+^ signal (Fig. 5b)^31^. Using a longer light pulse, the waveform of the Ca^2+^ signal changed in two respects: the amplitude of the initial Ca^2+^ signal became much smaller and, unexpectedly, a second Ca^2+^ signal was generated precisely when the photolyzing light was switched off. In keeping with our nomenclature, we refer to the first and second Ca^2+^ signal as ‘on' and ‘off' Ca^2+^ response, respectively.

To elucidate the cellular mechanism underlying the Ca^2+^ ‘off response', we probed the cGMP-induced voltage response using the same light protocol. Light stimulation produced a hyperpolarization^25^ (Fig. 5b). For both brief and long light pulses, the waveform of the rising phase of the hyperpolarization was identical. However, for long light pulses (that is, with prolonged cGMP ‘synthesis'), the decline from the hyperpolarizing peak was slower and incomplete; after the light was switched off (that is, when cGMP production ceased), the recovery from hyperpolarization was rapidly completed. The rapid *V*_m_ drop coincided with the onset of the Ca^2+^ ‘off response'. The following model readily accounts for these observations^22^,^26^: Voltage-dependent CatSper channels open during recovery from hyperpolarization. While the photolyzing light is on, cGMP does not return to baseline levels as quickly and completely as compared with a brief light pulse. Consequently, a fraction of CNGK channels are kept open, and sperm stay partially hyperpolarized. When cGMP production by light ceases, cGMP levels rapidly drop due to PDE activity. The rapid return to resting *V*_m_ opens additional CatSper channels and, thereby, produces the Ca^2+^ ‘off response'. The behavioural ‘off response' triggered by this Ca^2+^ signal results in a major correction of the swimming path. Along the new direction of swimming, sperm experience again an ascending chemoattractant concentration in time.

### Model of chemotactic steering

**Figure 6:**
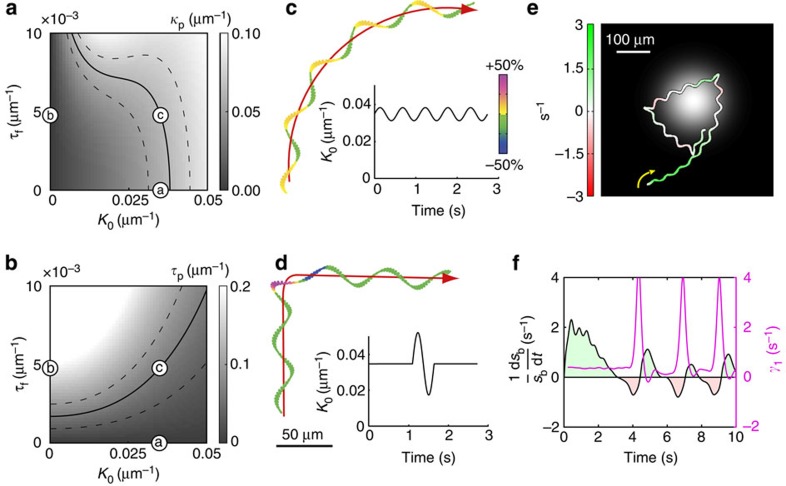
Theoretical model for sperm steering. (**a**,**b**) Path curvature (*κ*_p_; **a**) and torsion (*τ*_p_; **b**) predicted by theory as a function of the mean flagellar curvature *K*_0_ and flagellar twist *τ*_f_. The range of experimentally measured values is shown as contour lines (solid: mean, dashed: mean±s.d.). Labelled circles correspond to the beat patterns used in Fig. 2a–c. Only one pair (*K*_0_=0.035 μm^−1^, *τ*_f_=4.8 × 10^−3^ μm^−1^) produces a helical path with the observed mean path curvature and path torsion (encircled c). (**c**) Computed swimming path (left) resulting from modulating *K*_0_ at the frequency of helical swimming. The colour coding and the graph to the right show *K*_0_. Smooth bending of the helical path accounts for gradual ‘on responses'. The helical centre line is shown in red. (**d**) A pulse-like change of *K*_0_ accounts for sharp helix turns during ‘off responses'. (**e**) Computed path of a cell navigating in a chemoattractant gradient (grey shades), where *K*_0_ is dynamically adjusted by a simple steering feedback (combining a response to fast stimulus oscillations and a dynamic regulation of feedback strength by slow changes of the stimulus baseline, see Supplementary Note 1). (**f**) Relative change in baseline stimulus and alignment rate (*γ*_1_) for the simulated path shown in **e**.

Previous theoretical models did not address how the flagellar beat steers a cell in a gradient^15^,^19^. Moreover, they predicted steady adjustment of the swimming path by a constant feedback mechanism. We developed further the theory of chemotactic steering in two respects: a dynamic regulation of flagellar waveform and an adaptive steering feedback. For steering, sperm must alter the waveform of their flagellum. Therefore, we first computed helix parameters for constant waveforms (characterized by mean flagellar curvature *K*_0_ and constant flagellar twist); this allowed the identification of a unique parameter set that reproduces the helix geometry in the absence of stimulation (Fig. 6a,b). Furthermore, 2D experiments suggest that a chemotactic signalling pathway regulates the mean flagellar curvature^9^,^12^. Therefore, we simulated the 3D swimming path resulting from variations of *K*_0_. A small-amplitude, periodic modulation of *K*_0_ causes steady helix bending (Fig. 6c), whereas a single large-amplitude modulation elicits a sharp turn (Fig. 6d). Helix bending and sharp turn are reminiscent of the ‘on' and ‘off response', respectively. Second, in an extended model of chemotactic steering, *K*_0_ is dynamically adjusted to the stimulus concentration sampled by the swimming cell (see Supplementary Note 1). For up the gradient swimming, when only small course corrections are needed to align the helix, the signalling feedback strength is small; consequently, the amplitude of *K*_0_ oscillations is small too, resulting in steady alignment (‘on responses'). If, however, sperm swim down a gradient, an upregulated feedback strength allows for sharp turns (‘off responses'). Simulations using this generic model account for the chemotactic ‘on' and ‘off' steering responses and show robust chemotaxis towards the summit of a resact concentration profile (Fig. 6e,f).

## Discussion

We identify the principal features of sperm navigation in a 3D chemical landscape. Sperm probe the chemoattractant concentration along helical paths and, thereby, a spatial gradient is translated into a temporal stimulus pattern that oscillates with the 2 Hz periodicity of helical movement. Thus, the swimming path organizes the temporal stimulus pattern perceived by sperm, a principle known as information self-structuring^41^. The periodic component is superimposed on a mean stimulus level (baseline) that either increases or decreases slowly when swimming up or down a continuous gradient, respectively. The rapid stimulus oscillations provide a sense of direction, whereas the baseline slope controls the response strength. A positive slope signifying a chemoattractant increase produces weak ‘on responses'; a negative slope when losing track evokes strong ‘off responses'. This regulation of response strength allows sperm to tune klinotaxis behaviour ranging from subtle helix bending to abrupt emergency turns.

Response regulation can be achieved by dynamic adjustment of feedback strength, which is superior to a previous model with only constant feedback^19^. What might be the cellular signalling mechanisms underlying dynamic feedback strength? The cGMP dynamics, resulting from cGMP degradation by PDE activity and inactivation of the receptor guanylyl cyclase^27^, controls the recovery from hyperpolarization and, thereby, the amplitude and timing of Ca^2+^ responses. Based on our macroscopic Ca^2+^ measurements, we propose a model in which slowly increasing stimulation during helix alignment keeps cGMP levels elevated and the recovery from hyperpolarization is, therefore, slower and incomplete; consequently, Ca^2+^ responses turn out small. When stimulation is waning while drifting down the gradient, cGMP hydrolysis outcompetes cGMP synthesis. As a result, CNGK channels close swiftly and *V*_m_ rapidly drops to resting values, thus triggering a large ‘off' Ca^2+^ signal that drives an ‘off response'. We propose that the regulated feedback strength resides in the dynamic balance between cGMP synthesis and hydrolysis. Future work needs to combine holographic microscopy with fluorescence imaging techniques to monitor the signalling events in single cells during 3D chemotaxis.

While sperm from many marine invertebrates reach the egg by free 3D swimming, sperm from some fish swim on the 2D surface of the large egg during their search for a small fertilization site, known as the micropyle^42^. Moreover, mammalian sperm swim along the narrow oviduct and intermittently interact with the convoluted epithelium that lines the oviduct^43^, thus mammalian sperm probably switch between 2D and 3D navigation. How similar are 2D and 3D navigation mechanisms? For sea urchin sperm, the drifting-circle pattern observed during 2D chemotaxis on a surface is equivalent to the helix-bending mode of 3D klinotaxis. Because the ‘off response' is inherently rooted in the design of cellular signalling, we expect that the control logic is similar for 2D and 3D scenarios. Thus, why have ‘off responses' escaped detection in studies of 2D navigation? For 2D swimming at interfaces, movements are constrained and subject to hydrodynamical interactions with the surface. Consequently, the ballistic component of movement along the helical path is missing because movement across the surface is not possible. The degree of freedom for 3D swimming is manifold larger and thus is the likelihood that sperm eventually will swim away from the chemical source. Therefore, the ‘off response' is more important and prominent for 3D than for 2D swimming. Future work needs to address the behavioural ‘off response' under 2D conditions, for example, by applying the approach of reversed optochemical engineering at the single cell level.

How common are navigation strategies of sperm across phyla? For mammalian sperm, three different mechanisms of guidance have been proposed: chemotaxis^44^,^45^, thermotaxis^46^,^47^ and rheotaxis^4^,^48^. Human sperm, when rapidly stimulated with progesterone, a putative chemoattractant, released from caged progesterone^49^ undergo hyperactive episodes that change the swimming direction^45^. Similar events are observed in a thermotaxis assay after a temperature shift to lower values^47^. Superficially, these hyperactive episodes observed in human sperm are reminiscent of the ‘off responses'. However, unlike ‘off responses', they initiate a stochastic rather than deterministic reorientation and are more akin to stochastic tumbling episodes of bacterial chemotaxis.

Klinotaxis is a basic mechanism of navigation displayed by many motile cells and organisms^50^, including the nematode *Caenorhabditis elegans*, larva from *Drosophila*, *Platynereis* and zebrafish primordial germ cells, protists and even some bacteria^51^,^52^,^53^,^54^,^55^. Sensory modalities as diverse as phototaxis, thermosensation, taste, olfaction and electroreception employ klinotaxis. In many animals, neuronal circuits are dedicated to analyse the temporal pattern of sensory stimuli that instruct the klinotactic response. Moreover, neuronal circuits are composed of ‘on' and ‘off cells' that register the ups and downs of sensory stimuli. Temporal sampling during klinotaxis is either accomplished along a periodic path or by undulatory movements of the body. It is quite remarkable that a signalling pathway can encompass these computational features in a single cell.

On a final note, chemoattractant landscapes around an egg in a natural habitat are not known and are expected to be rather complex. Local chemoattractant concentrations might be rapidly changing and distorted by turbulent fluid flow and drifting of eggs, giving rise to ‘plumes' rather than Gaussian-shaped continuous gradients^56^. To emulate these native conditions and to study how sperm, and any other microswimmers, cruise in such complex chemical landscapes is the next frontier of enquiry. The optochemical and microscopy techniques presented here combined with computational models provide the means to generate and quantify complex landscapes and to reveal the search strategy during microswimmer navigation in general.

## Methods

### Sample preparation

Sperm spawning was evoked by injecting 0.5 ml of 0.5 M KCl into the body cavity of sea urchins from the species *A. punctulata*. Sperm were collected using a Pasteur pipette and stored on ice. Artificial seawater (ASW) contained (in mM) 423 NaCl, 9.27 CaCl_2_, 9 KCl, 22.94 MgCl_2_, 25.5 MgSO_4_, 0.1 EDTA and 10 HEPES. The pH was adjusted to pH 7.8 with NaOH.

### Imaging chamber

Dry sperm were diluted 6.6 × 10^4^ fold with ASW supplemented with 0.5% Pluronic F127 (Sigma-Aldrich) and studied in a custom-made observation chamber (11.5 × 11.5 × 1 mm^3^). The chamber featured two openings for sperm injection. To minimize convective flow, the laboratory and the microscope incubator were set to 18 °C. The temperature of 18 °C was chosen because it roughly corresponds to the average temperature of the northern Atlantic coast during the fertility season of *A. punctulata*^57^.

### Optical set-up

Freely swimming sperm were tracked using an inverted microscope (IX71; Objective × 20, 0.75 numerical aperture, UPLSAPO; Olympus). Coherent illumination was achieved with a laser light source (LDH-D-C-510, PicoQuant GmbH) and the corresponding controller (Sepia II Multichannel Processor, PicoQuant GmbH). Laser light was coupled into a multimode fibre. A custom-made adapter was used to position the fibre parallel to the optical axis of the objective. The illumination intensity was adjusted to use the dynamical range of the camera (12-bit; PCO Dimax HD). Movies were collected at 600 frames per second; each frame represents a holographic image containing the complete 3D information of the sperm cell.

Caged compounds were photolyzed using a 365-nm LED (M365L2-C; Thorlabs). The ultraviolet light was coupled into a liquid guide (77566; Newport) followed by two Plano-convex lenses (LA 1951-A *f*=25.4 mm; LA 1509-A *f*=100 mm; Thorlabs) and coupled to the imaging optical path with a dichroic filter (ff 495-Di03; Semrock). The irradiation power (0.8 mW) was measured with a power meter (detector PowerMax and head model PS19Q; Coherent). The light spectrum was recorded with a spectrometer (51024 DW; Ocean Optics). Photolysis and data acquisition were synchronized using a wave generator (33500B; Agilent).

### Reconstruction of the 3D swimming path

The 3D swimming path was reconstructed in several steps. First, we created a ‘background' image from the average intensity value of the hologram sequence (usually 24,000 frames). Because moving objects in the hologram sequence are averaged out, the background image contains only the non-scattered light and the fringes resulting from interference of the source with non-moving particles. Dividing each hologram by the background image results in distinct interference patterns of moving sperm^32^. To curb the data volume, single sperm cells were tracked within the plane of the image using a custom-made tracking programme written in Java (Java 1.6 24; ImageJ v.1.47 m). The determined position was used to define a moving region-of-interest (ROI) of typically 300 × 300 pixels around the sperm cell for each frame; this ROI was used for further analysis.

The Rayleigh–Sommerfeld back-propagation scheme was used to numerically refocus each background-free hologram. Numerical refocusing resulted in a focus stack of computed images. The *z*-position of the cell corresponded to the point of contrast inversion (Gouy phase anomaly^58^; Supplementary Fig. 1). Contrast inversion was identified by an image-processing filter that highlights axial gradients in the image stack, such as the Sobel filter^33^,^59^,^60^.

### Caged compounds and flash photolysis

N-Fmoc-S-(2-Nitroveratryl)-L-cysteine was obtained as described^61^ and used for solid-phase peptide synthesis (Biosyntan GmbH, Berlin, Germany) to obtain caged Cys8-S-DMNB-resact^31^. The extinction coefficient of caged resact (20 μM) in ASW was measured by an absorption spectrophotometer (Varian Carry 5000). To determine the photochemical quantum yield (*φ*_chem_), a 50 μM solution of the caged peptide in ASW was irradiated with a 365±5 nm ultraviolet-LED providing an power flux of 90 mW cm^−^^2^ (NCSU033A(T), Nichia, Japan). At several time points aliquots were taken and analysed via HPLC (*n*=3). From the initial slope of linear release (0–15% conversion) and the rate of photon absorption, determined by ferrioxalate actinometry^62^, we determined a photochemical quantum yield of *φ*_chem_=0.4±0.1%. DEACM-caged cGMP was synthesized as described^63^.

### Characterization of the 3D gradient

The ultraviolet-light profile was characterized by measuring optical sections along the *z*-axis. A stack of seven glass cover-slides (150-μm thickness each) was placed at the location of the observation chamber. The ultraviolet profile was measured between slides by adding fluorescein (10 μl; 1 mM) and imaging the fluorescence emission (emission filter ET 510 LP; Chroma) that resulted from excitation of fluorescein using the LED ultraviolet source. Because the geometry of the spatial light profile is fixed with respect to the objective lens, focusing on the individual planes was achieved by moving the camera to the corresponding conjugate plane. The resulting image was rescaled to compensate for the change in magnification. The scaling factor was measured by imaging a grid (DV2 calibration grid; Photometrics) at each camera position. Individual fluorescence pictures, corresponding to the ultraviolet projection on the different planes, were fitted to a Gaussian function (*R*^2^>0.977). The change in width along the different optical sections was well-described by a Gaussian beam^64^ (Supplementary Fig. 2).

To calculate the resact concentration in time and space, we numerically solved Fick's second law in 3D using the Euler method for both resact and caged resact. Space was discretized on cubes of 5-μm side, and the integration time step chosen was 1.67 ms. The diffusion of resact^39^ (*D*_resact_=239 μm^2^ s^−1^) and caged resact were assumed to be identical. The release of resact from its caged analogue (100 nM caged resact) was calculated on each iteration based on the power and spectrum of the ultraviolet source, the extinction coefficient (*ɛ*=4,100 M^−1^ cm^−1^) and the photochemical quantum yield of DMNB-caged resact. Programmes were written in MATLAB (Mathworks).

### Quantification of the helical swimming path

From the tracked sperm head trajectory, we define an averaged path **r**(*t*) by averaging out wiggling of the sperm head at the frequency of the flagellar beat, using a least-square fit that simultaneously minimizes the average disance of **r**(*t*) to the head trajectory and the third time derivative of track coordinates. The implicit prior on the third spatial derivative ensures smoothness of the averaged path without introducing a bias on the path curvature. We then fitted perfect helices to short **r**(*t*) segments using sliding windows of 0.75 s. This defines a helical path centre line **R**(*t*), and the helix vector **h**=d**R**/d*t*/|d**R**/d*t*|.

We define *s*(*t*)=*c*(**r**(*t*),*t*) as a proxy for the concentration stimulus sampled by the cell in a concentration field *c*(**x**,*t*) and compute the local gradient direction ∇*c*(**R**,*t*). This gradient can be decomposed into components parallel (∇_||_*c*=(∇*c*·**h**)**h**) and perpendicular (∇_⊥_*c*=∇*c* −∇_||_*c*) to the helix vector (Fig. 3c). If the helix were perfectly aligned with the gradient direction, ∇_⊥_*c* would be zero.

We characterize helix bending (d**h**/d*t*) with respect to the local gradient direction ∇*c* as


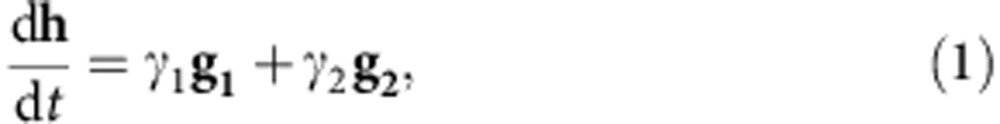


where **g**_**1**_=∇_⊥_*c*/|∇_⊥_*c*|, **g**_**2**_=**h** × **g**_**1**_ and **h** form a local coordinate system that moves along the helix centre line. Thus, aligment occurs when the helix bends towards **g**_**1**_ (*γ*_1_>0). Any bending in the direction **g**_**2**_ does not contribute to chemotaxis. For the histogram showing the direction of helix bending relative to the gradient direction (Fig. 3c), we analysed *n*=10 cells along their full tracks every 0.75 s, corresponding to 152 data points.

To compute path curvature *κ*_p_(*t*) and torsion *τ*_p_(*t*) of **r**(*t*), we fitted perfect helices to sliding path segments of duration 0.25 s. To stabilize the nonlinear helix fitting algorithm, we included a prior to the cost function that penalizes strong deviations from the helix parameters obtained for fitting a helix to a longer path segment of 0.75-s duration. The path curvature and path torsion were obtained from the radius and pitch of the fitted helical segments. We define the oscillatory part of *s*(*t*), *κ*_p_(*t*) and *τ*_p_(*t*) by subtracting a baseline defined by a Fourier filter with cut-off frequency at 1 Hz. To determine the phase-shifts (*φ*_*κ*_ and *φ*_*τ*_) between stimulus and curvature/torsion oscillations, we fitted a model Acos(Ω_0_Δ*t*+*φ*)e^−*D*Δ*t*^ of phase-locked oscillations to their normalized cross-correlation, where Ω_0_ is the helix frequency and *D* accounts for phase diffusion.

To characterize the fraction of cells displaying ‘off responses', we selected those cells for which at least 3,000 frames (equivalent to 5 s) had been successfully tracked using our 3D tracking algorithm. ‘Off responses' were attributed to alignment events where the angle between the helical axis and the gradient abruptly changed from values exceeding 90°.

We determined the plane of head wiggling by fitting planes to short segments of the head trajectory of 150-ms duration, using singular-value-decomposition on centred coordinate data.

### Numerical simulations of swimming sperm

We numerically reconstructed the swimming path that would result from prototypical flagellar waveforms using resistive-force theory^35^,^36^. Specifically, we computed the anisotropic hydrodynamic friction forces associated with shape changes of the slender flagellum. This allowed us to self-consistently solve for the instantaneous motion of the cell's material frame under conditions of force balance. Instantaneous translations and rotations were integrated using an explicit Euler scheme and the formalism of rigid body transformations. The time-dependent shape of the flagellum was characterized by the position vector **r**_f_(*l*,*t*) of the flagellar centre line as a function of arc length *l* along the flagellum and time *t*, as well as a Cosserat frame consisting of three orthonormal vectors **e**_1_(*l*,*t*), **e**_2_(*l*,*t*) and **e**_3_(*l*,*t*), such that **e**_3_ is tangential to the flagellar centre line **r**_f_, and **e**_1_ and **e**_2_ span a normal cross-section of the flagellum. Bending and twisting of the flagellum are characterized by rotations of the Cosserat frame:


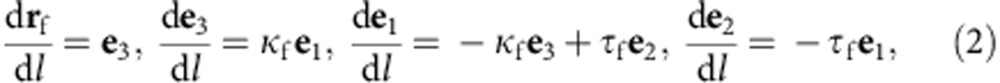


where *κ*_f_(*l*,*t*) denotes flagellar curvature and *τ*_f_ denotes flagellar twist. We assume a constant flagellar twist *τ*_f_ and a flagellar curvature *κ*_f_ given by a travelling bending wave,


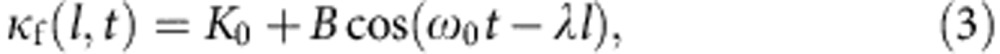


where *K*_0_ is mean curvature, *B* the amplitude, *ω*_0_ the angular frequency and 2*π*/*λ* the wavelength of the flagellar bending waves.

We assumed parallel and normal flagellar friction coefficients *ξ*_||_=0.99*η*, and *ξ*_⊥_=1.81*ξ*_||_, respectively, where *η* denotes the dynamic viscosity of the surrounding liquid^35^. The flagellar length (41 μm), flagellar wavelength (2*π*/*λ*=29.6 μm) and amplitude of the flagellar curvature wave (*B*=0.160 μm^−1^) were estimated from flagellar tracking of sperm swimming close to a water/glass interface. The beat period (2*π*/*ω*_0_=23 ms) was estimated from the frequency of head wiggling for sperm swimming in 3D. Mean flagellar curvature (*K*_0_=0.0351 μm^−1^) and flagellar twist (*τ*_f_=0.00477 μm^−1^) were obtained by a nonlinear fit to reproduce the experimentally measured helix parameters, see Fig. 5a,b. The sperm head was approximated by an ellipsoid with axes 2.5, 1.25 and 1.25 μm, as estimated from electron micrographs. Finally, the proximal end of the flagellum (neck) is assumed collinear with the long axis of the sperm head.

### Numerical simulation of sperm chemotaxis

Using our hydrodynamic simulation scheme, we computed sperm swimming paths **r**(*t*) in a concentration landscape *c*(**x**,*t*) of chemoattractant. We assume a dynamic regulation of the shape of the flagellar beat in response to the concentration stimulus *s*(*t*) sampled by the cell along its path:


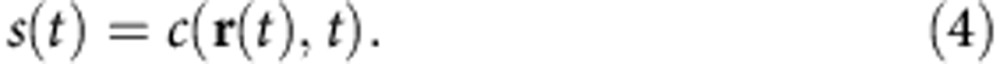


The time-dependent stimulus *s*(*t*) is transduced by a simple adaptation module^19^,^65^,^66^, where *p*(*t*) denotes a dynamic sensitivity, *a*(*t*) the signalling output variable and *μ* a signalling time-scale:


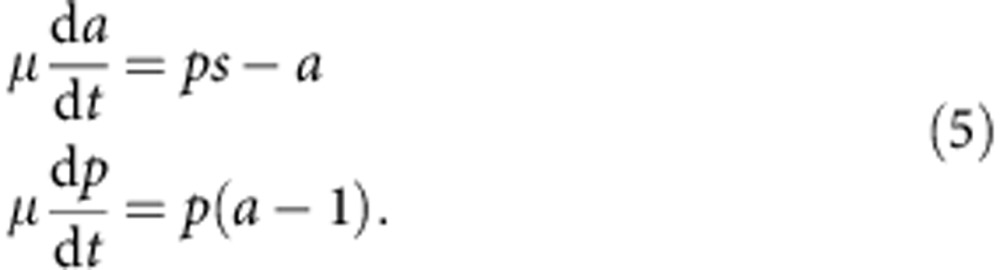


This minimal description embodies key characteristics of the sperm chemotactic signalling system as observed in experiments, namely an adaptation of sensitivity according to the baseline stimulus level^39^, as well as relaxation to the rest state (*a*=1) for any stimulus that does not change in time^9^. Further characteristics of the signalling module (equation (5)) are provided in the Supplementary Information text. The output variable *a*(*t*) regulates the mean flagellar curvature *K*_0_ of the flagellar beat according to:


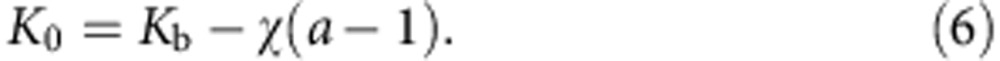


Here *K*_b_ denotes the mean flagellar curvature of unstimulated sperm and *χ* denotes a feedback strength. For simplicity, all other parameters of the flagellar beat are assumed constant. A dynamic regulation of mean flagellar curvature on chemotactic stimulation has been demonstrated in previous 2D experiments^12^.

To conceptualize our novel experimental finding of ‘off responses', we employ a dynamic feedback strength *χ*(*t*) that can alternate between a low (*χ*_on_) and a high value (*χ*_off_) for steady swimming up or down the gradient, respectively. We introduce a signalling variable *q*(*t*), which tracks changes of the stimulus baseline, and that obeys the following low-pass filter dynamics:


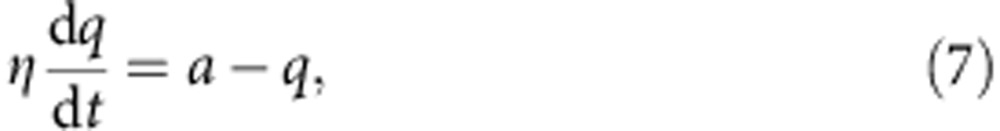


where *η* denotes a relaxation time-scale. In our minimal description, the dynamic feedback strength *χ*(*t*) takes either of the two values, depending on whether *q*(*t*) exceeds a threshold *θ*:


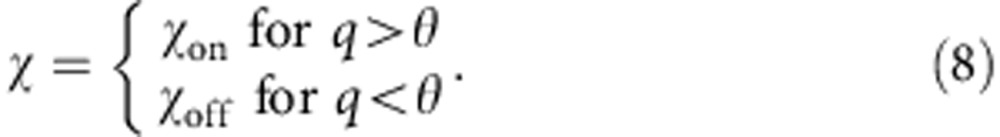


For the simulations, we employed the concentration landscape *c*(**x**,*t*) corresponding to the experiment shown in Fig. 3b. Parameters: *μ*=150 ms, *η*=500 ms, *θ*=0.95, *χ*_on_=*K*_b_, *χ*_off_/*χ*_on_=8 and *K*_b_=0.0351 μm^−1^.

### Measurement of changes in [Ca^2+^]_i_ and membrane voltage *V*_m_

We measured changes in [Ca^2+^]_i_ and *V*_m_ using a rapid-mixing device (SFM-400; Bio-logic) in the stopped-flow mode. Changes in [Ca^2+^]_i_ and *V*_m_ were measured with the Ca^2+^ indicator Fluo-4 AM and the voltage-sensitive indicator di-8-ANEPPS (Molecular Probes), respectively. Dry sperm were suspended 1:6 (vol/vol) in loading buffer containing ASW and the indicator, in the absence (di-8-ANEPPS) or presence (Fluo-4 AM) of 0.5% Pluronic F127 (Molecular Probes). After incubation for at least 45 min with Fluo-4 AM or 5 min for di-8-ANEPPS at 17 °C, the sample was diluted 1:20 with ASW. Sperm were allowed to equilibrate in the new medium for 5 min. In the stopped-flow device, the sperm suspension was rapidly mixed 1:1 (vol/vol) with resact in ASW or ASW alone. Concentrations of resact are given as final concentrations after mixing. Fluorescence was excited by a SpectraX Light Engine (Lumencor). Emission was recorded by photomultiplier modules (H9656-20; Hamamatsu Photonics). The signal was amplified and filtered through a voltage amplifier (DLPVA-100-B-S; Femto Messtechnik). Data acquisition was performed with a data acquisition pad (PCI-6221; National Instruments) and Bio-Kine software v. 4.49 (Bio-Logic). For Ca^2+^ and *V*_m_ recordings, the excitation light was passed through a BrightLine 475/28 nm filter (Semrock) (SpectraX Light Engine). For Ca^2+^ measurements, the emitted light was passed through a BrightLine 536/40-nm filter (Semrock). Ca^2+^ signals represent the average of at least two recordings and are depicted as the per cent change in fluorescence (Δ*F*) with respect to the mean of the first 5–10 data points before the onset of the signal (*F*_0_). The control (ASW) Δ*F*/*F*_0_ signal was subtracted from the resact- or cGMP-induced signals. The *V*_m_ signals were recorded in dual-emission mode. The filters in front of the two photomultipliers were BrightLine 536/40 nm and BrightLine 628/40 nm (Semrock). The Bio-Logic software was used to record the fluorescence in the ratiometric dual-emission mode. The *V*_m_ signals are the ratio of F536/628 or *R*. The control (ASW) *R* signal was subtracted from the resact- or cGMP-induced signals. The mean *R* of the first 5–10 data points before the onset of the changes in fluorescence was set to 0, yielding Δ*R*. The *V*_m_ signals represent the average of at least three recordings and were digitally smoothed with five-point average smoothing. The changes in di-8-ANEPPS fluorescence were calibrated into *V*_m_ (mV) by stimulating sperm with 2.5 nM resact concentrations in ASW (9 mM *K*^+^) and ASW of 30 mM and 100 mM extracellular K^+^. Plotting the resact-evoked Δ*R* against [*K*^+^]_o_ allowed the interpolation of *V*_rest_ and Δ*R*/mV as previously described^25^. Calibration of Δ*R* to mV was performed within each set of experiments. The data obtained from ensemble measurements were analysed using OriginPro 9.0 (OriginLab Corporation).

### Data analysis

All data are given as mean±s.d. unless otherwise stated.

## Additional information

**How to cite this article:** Jikeli, J. F. *et al*. Sperm navigation along helical paths in 3D chemoattractant landscapes. *Nat. Commun.* 6:7985 doi: 10.1038/ncomms8985 (2015).

## Supplementary Material

Supplementary Information Supplementary Figures 1-4, Supplementary Note 1 and Supplementary References

Supplementary Movie 1 Gaussian UV light beam used for photolysis. The vertical grey line indicates the centre of the photolyzing beam along the optical axis.

Supplementary Movie 2 Reconstructed chemoattractant landscape. The chemoattractant concentration (in nM) is color-coded. The vertical grey line indicates the centre of the photolyzing beam along the optical axis.

Supplementary Movie 3 Reconstructed swimming path of a sperm cell in a gradient. The vertical grey line indicates the centre of the photolyzing beam along the optical axis. Red arrowheads indicate abrupt chemotactic turns. The swimming path unfolds at half the real speed.

Supplementary Movie 4 3D Anaglyph of the reconstructed swimming path of a sperm cell in a gradient. The vertical grey line indicates the centre of the photolyzing beam along the optical axis. Red arrowheads indicate abrupt chemotactic turns. The swimming path unfolds at half the real speed. The movie should be watched with 3D red cyan glasses.
