# Supplementary material for: Sperm navigation along helical paths in 3D chemoattractant landscapes
Source: Nat Commun. 2015 Aug 17;6:7985. doi: 10.1038/ncomms8985 (PMC4557273; doi:10.1038/ncomms8985)
Supplement: Supplementary Information — Supplementary Figures 1-4, Supplementary Note 1 and Supplementary References [file ncomms8985-s1.pdf]

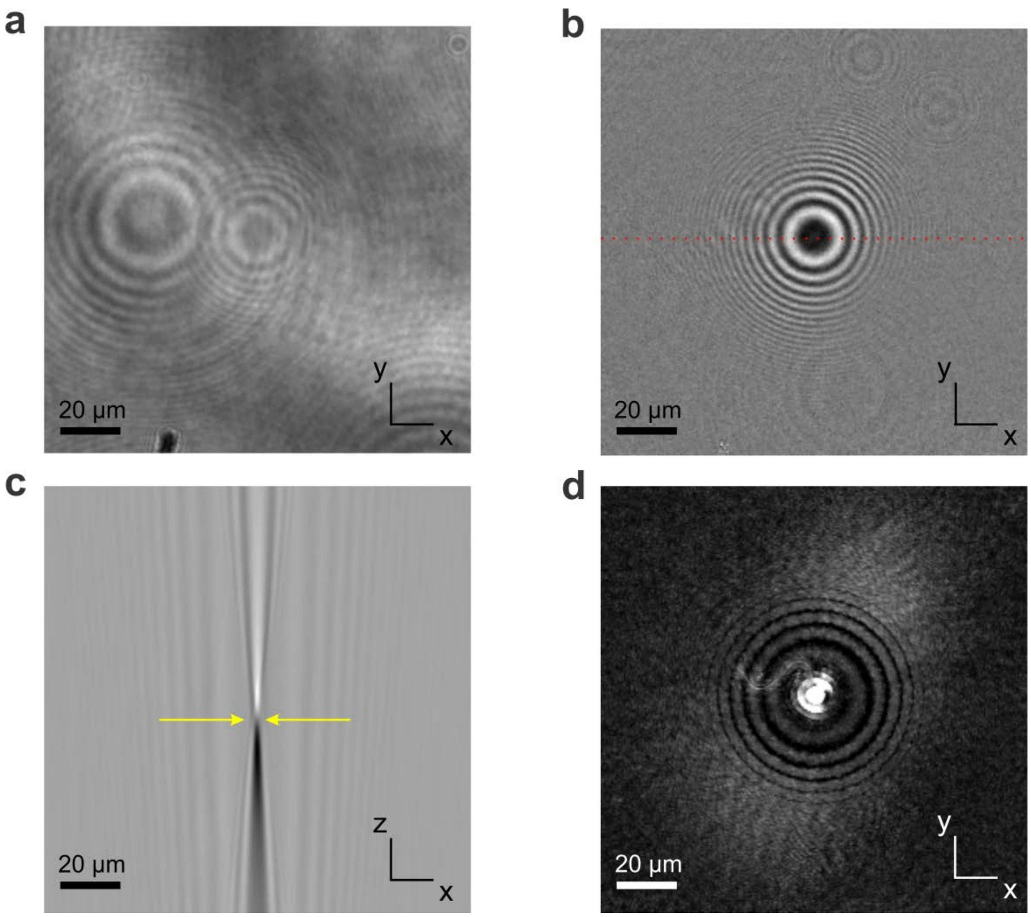

Supplementary Figure 1: Tracking of sperm head from individual holograms. (**a**) A frame of raw holographic data; the sperm head can be seen as the fainter series of concentric diffraction fringes at the centre of the image. (**b**) A frame of background-corrected holographic data (see methods). The centroid of the circular diffraction pattern provides the x-y position of the head. From background-corrected holograms, a refocused image stack was created. (**c**) Section through the refocused stack at a fixed y-position. The position is indicated by the red dashed line in (b). The z-coordinate of the sperm head is derived from the steepest intensity gradient along z (the point of contrast inversion is indicated by yellow arrows). (**d**) A portion of the refocused stack around the cell was extracted and a gradient filter was applied to highlight axial intensity gradients. This gradient stack was projected along the z-direction, retaining the maximum intensity value at each x-y position. A faint image of the flagellum becomes visible.

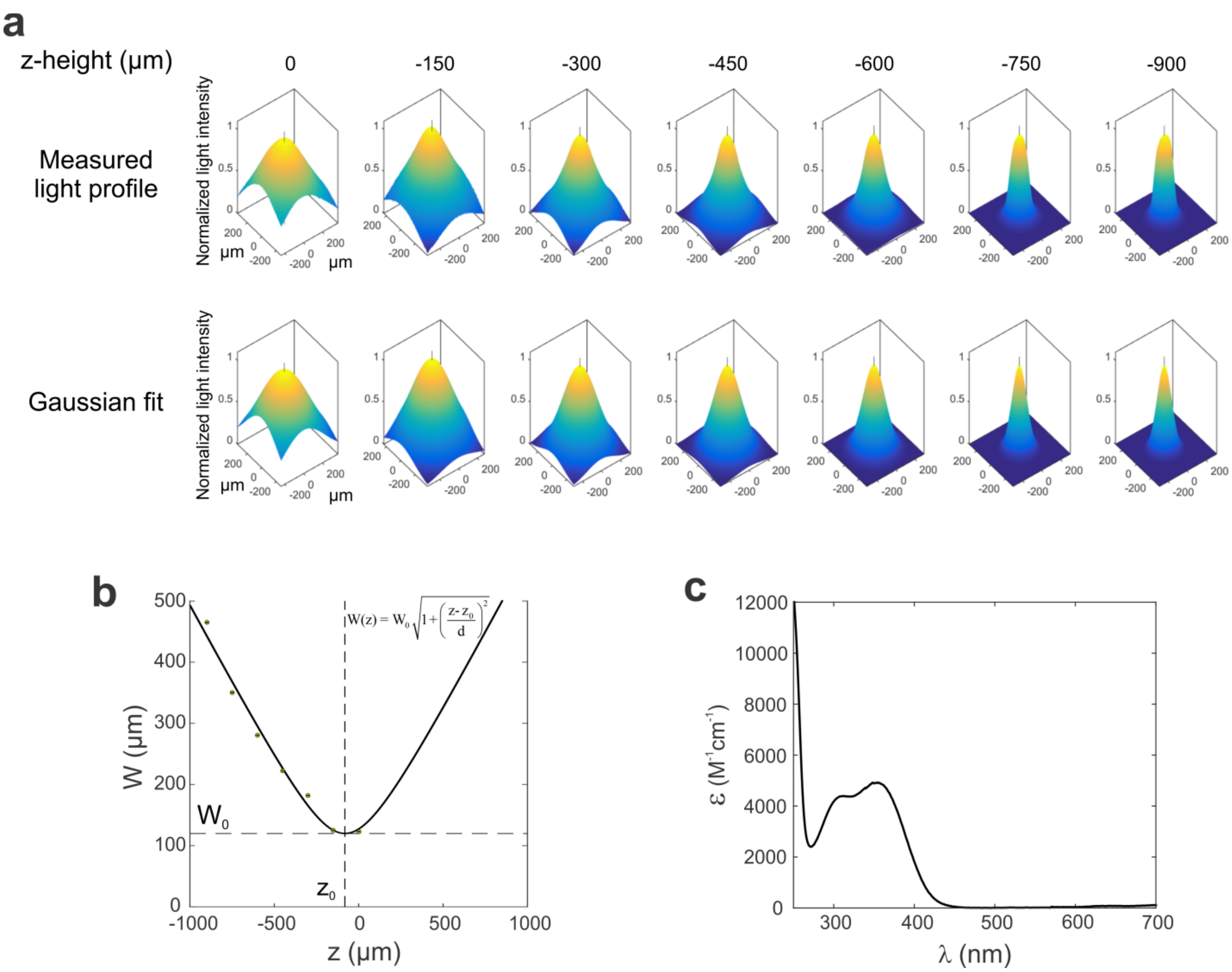

Supplementary Figure 2: Characterization of the chemoattractant release. (**a**) Normalized profile of UV light used for release of resact (top). The UV profile was measured by exciting fluorescein sheets distributed at different *z*-positions along the optical axis of the microscope. The focal plane of the objective corresponds to $z = 0$ µm. Individual sections were fitted to a Gaussian function (bottom). (**b**) Fitted width of the UV light profile at different heights along the optical axis (data in points yellow with black outline; thin black error bars lay within the points). The width of the light at different *z*-positions resembles a Gaussian beam (equation, and corresponding fit shown as a thick black line). (**c**) Molar extinction coefficient of caged resact in ASW.

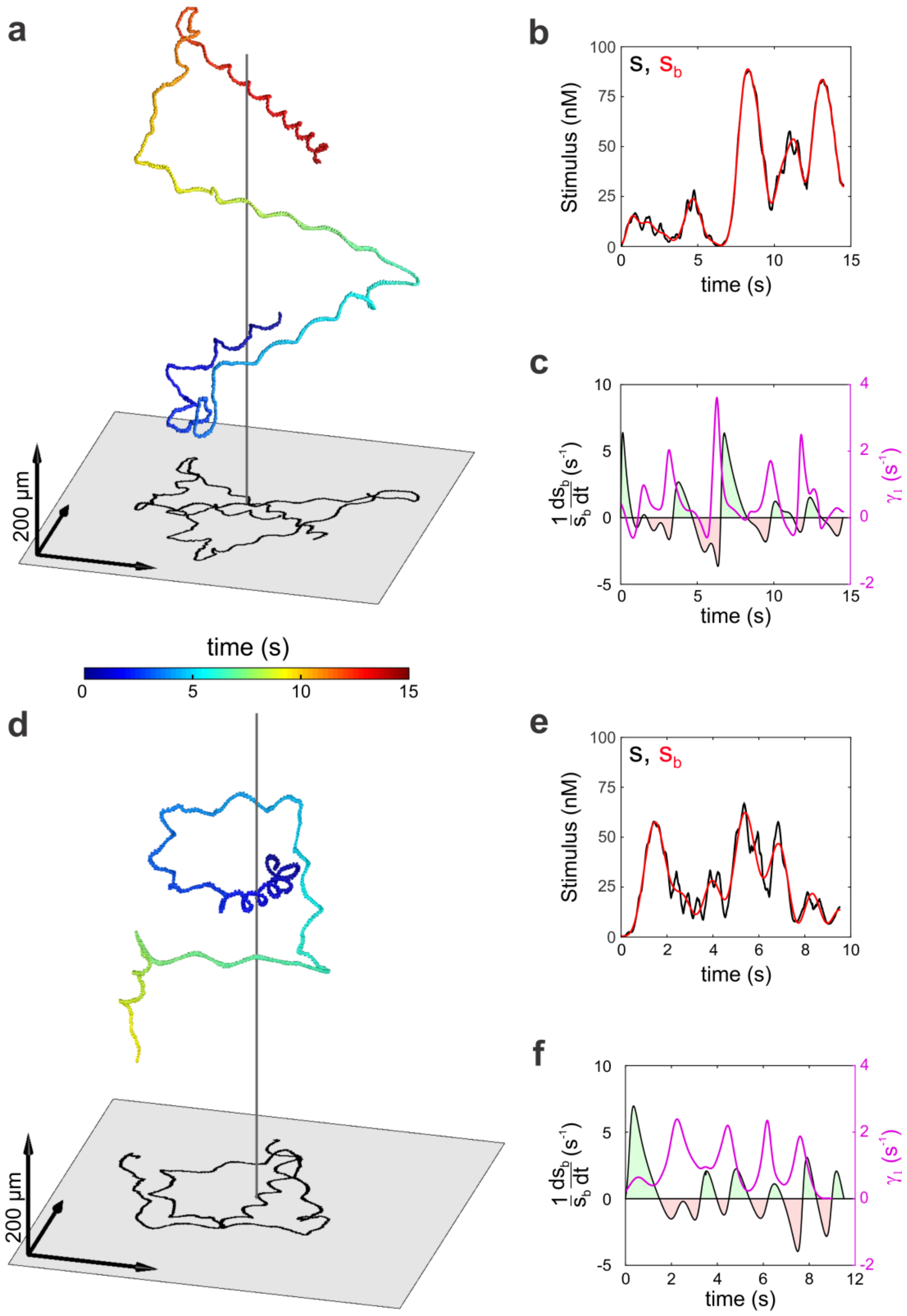

Supplementary Figure 3: Sperm steering in a 3D gradient. (**a, d**) representative swimming paths of sperm in the gradient shown in Figure 3a. (**b**, **e**) Corresponding stimulus encountered by sperm while swimming in the gradient. (**c, f**) Relative changes of the stimulus baseline and alignment of the helical path.

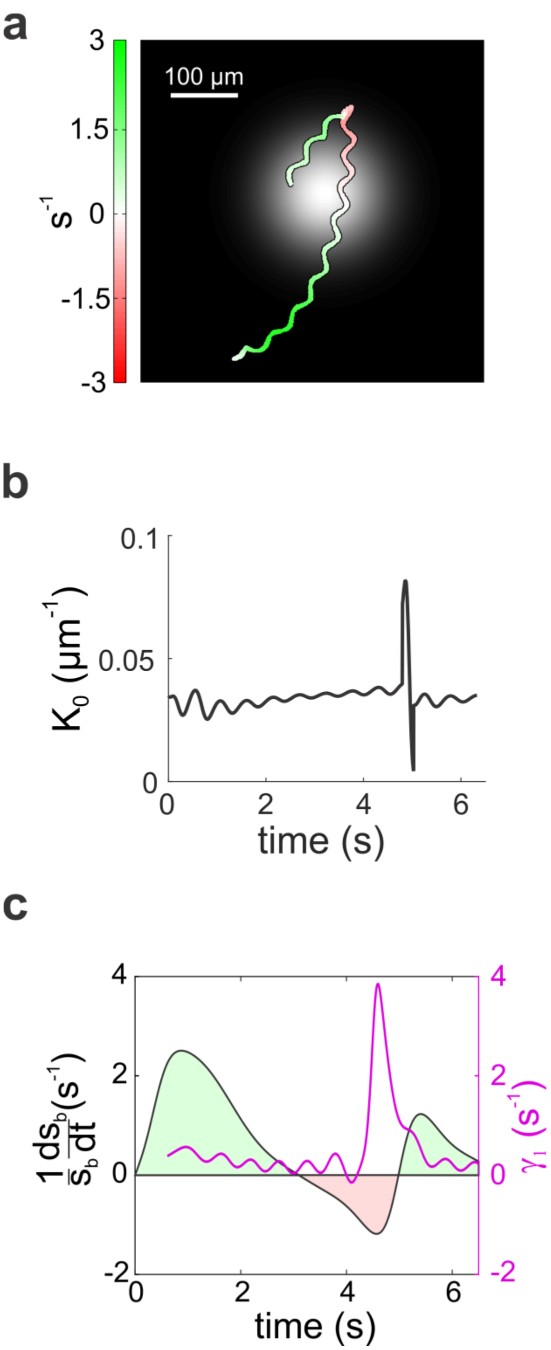

Supplementary Figure 4: Exemplary simulation of sperm chemotaxis when chemoreceptors are located along the flagellum. (**a**) Computed path of a cell navigating in a chemoattractant gradient (grey shades), where K_0 is dynamically adjusted by a simple feedback. The chemoattractant sensors have been assumed to be distributed uniformly along the flagellar surface (see Supplementary Note). Regardless of the chemoreceptor location (head versus flagellum) the navigation behaviour is analogous (see Fig. 6. and Supplementary Note). (**b**) Mean flagellar curvature in time K_0 for the simulated path shown in panel a. During swimming up the gradient, K_0 modulations are small. Swimming down the gradient triggers a large modulation of the mean flagellar curvature and results in a large correction of the swimming direction. (**c**) Relative change in baseline stimulus and alignment rate (γ_1) for the simulated path shown in panel a.

Supplementary Note

Jan F. Jikeli[1]*, Luis Alvarez[1]*, Benjamin M. Friedrich[2]*, Laurence Wilson[3]*, René Pascal[1], Remy Colin[4], Magdalena Pichlo[1], Andreas Rennhack[1], Christoph Brenker[5], and U. Benjamin Kaupp[1]: **Sperm navigation along helical paths in 3D chemoattractant landscapes**

Theoretical description of steering along helical paths

This SI text presents a theoretical analysis of our mathematical model of sperm chemotaxis along helical paths. We first summarize symbols and equations used. Second, we characterize the input-output transfer function of the signaling module for three prototypical stimulus functions $s(t)$. We then review the kinematics of swimming along perfect helical paths, as well as steering along bent helices by oscillating path curvature and path torsion. We specifically address *on* and *off* steering responses. We discuss the role of phase lags – both due to signaling latency as well as due to the dynamics of swimming – between stimulus and motor response.

List of symbols used:

t:	time
$\mathbf{x}$:	space coordinates
$\mathbf{r}(t)$:	averaged swimming path of the sperm cell
$c(\mathbf{x},t)$:	chemoattractant concentration-field
$s(t)$:	concentration stimulus sampled by the cell along its path
$\mathbf{R}(t)$:	centerline of helical swimming path $\mathbf{r}(t)$
$\mathbf{h}(t)$:	unit vector pointing along the helical axis of a helical swimming path $\mathbf{r}(t)$
$\nabla c(t)$:	chemical gradient at $\mathbf{r}(t)$
$\nabla_{\parallel} c(t)$:	component of the gradient parallel to $\mathbf{h}(t)$

$\nabla_{\perp} c(\mathbf{x},t)$:	component of the gradient perpendicular to $\mathbf{h}(t)$
$\mathbf{g}_1(t)$:	unit vector parallel $\nabla_{\perp} c(t)$
$\mathbf{g}_2(t)$:	unit vector perpendicular to $\mathbf{h}(t)$ and $\mathbf{g}_1(t)$
$\gamma_1(t)$:	alignment rate of the helix vector $\mathbf{h}(t)$ towards $\mathbf{g}_1(t)$
$\gamma_2(t)$:	alignment rate of the helix vector $\mathbf{h}(t)$ towards $\mathbf{g}_2(t)$
$p(t)$:	dynamic sensitivity of signaling system
$a(t)$:	signaling output variable
μ:	signaling time-scale
$\chi(t)$:	steering feedback strength
χ_{on}:	steering feedback strength during *on* resonse
χ_{off}:	steering feedback strength during *off* response
$q(t)$:	trigger variable that monitors changes of the stimulus baseline
θ:	trigger threshold for *off* responses
η:	time-scale of stimulus filtering
$v(t)$:	swimming speed
$\kappa_{\mathrm{p}}(t)$:	path curvature of swimming path $\mathbf{r}(t)$
$\tau_{\mathrm{p}}(t)$:	path torsion of swimming path $\mathbf{r}(t)$
$\mathbf{t}(t)$:	tangent vector of the Frenet frame along the swimming path $\mathbf{r}(t)$
$\mathbf{n}(t)$:	normal vector of the Frenet frame along the swimming path $\mathbf{r}(t)$
$\mathbf{b}(t)$:	binormal vector of the Frenet frame along the swimming path $\mathbf{r}(t)$
r_0:	helix radius
p_0, $2\pi h_0$:	helix pitch

Ω_0: angular frequency of helical swimming

$K_0(t)$: mean flagellar curvature

K_b: mean flagellar curvature in the absence of stimulation

l: arc-length coordinate along the flagellum

$\mathbf{r}_f(l,t)$: position of the flagellar centerline

$\kappa_f(l,t)$: flagellar curvature

τ_f: flagellar twist

ω_0: angular flagellar beat frequency

$2\pi/\lambda$: wavelength of flagellar bending wave

$\mathbf{e_1}(l,t)$: normal vector of the Cosserat frame along the flagellum

$\mathbf{e_2}(l,t)$: binormal vector of the Cosserat frame along the flagellum

$\mathbf{e_3}(l,t)$: tangent vector of the Cosserat frame along the flagellum

List of equations:

Helix bending:

$$\dot{\mathbf{h}} = \gamma_1 \mathbf{g_1} + \gamma_2 \mathbf{g_2} \tag{1}$$

(dot denotes time-derivative)

Flagellar wave form dynamics:

$$\frac{\mathrm{d}\mathbf{r}_\mathrm{f}}{\mathrm{d}l} = \mathbf{e}_3,\ \frac{\mathrm{d}\mathbf{e}_3}{\mathrm{d}l} = \kappa_\mathrm{f}\mathbf{e}_1,\ \frac{\mathrm{d}\mathbf{e}_1}{\mathrm{d}l} = -\kappa_\mathrm{f}\mathbf{e}_3 + \tau_\mathrm{f}\mathbf{e}_2,\ \frac{\mathrm{d}\mathbf{e}_2}{\mathrm{d}l} = -\tau_\mathrm{f}\mathbf{e}_1, \tag{2}$$

$$\kappa_f(l,t) = K_0(t) + B\cos(\omega_0 t - \lambda l) \tag{3}$$

Signalling dynamics:

$$s(t) = c(\mathbf{r}(t), t) \tag{4}$$

$$\mu \dot{a} = ps - a \tag{5.1}$$

$$\mu \dot{p} = p(a-1) \tag{5.2}$$

$$K_0 = K_b - \chi(a-1) \tag{6}$$

$$\eta \frac{\mathrm{d}q}{\mathrm{d}t} = a - q \tag{7}$$

$$\chi = \begin{cases} \chi_{\text{on}} & \text{for } q > \theta \\ \chi_{\text{off}} & \text{for } q < \theta \end{cases} \tag{8}$$

Characterisation of the adapation module

We discuss the input-output characteristic of the adaptation module in equations (5.1) and (5.2) for three prototypical stimuli $s(t)$.

Constant stimulus

For a constant stimulus of the form $s(t) = s_0$, the output $a(t)$ is always constant, $a(t) = 1$.

Oscillatory stimulus

An oscillating stimulus $s(t) = s_0 + s_1 \cos(\Omega t)$ with frequency Ω and (small) amplitude s_1 will elicit oscillations of the output variable around its rest value, $a(t) \approx 1 + \rho_\mathrm{a} s_1 \cos(\Omega t + \phi_\mathrm{a})$, with amplitude gain ρ_a and phase-lag ϕ_a. Here,

$$\rho_\mathrm{a} e^{i\phi_\mathrm{a}} = \frac{1}{s_0} \frac{i\Omega}{1 + i\Omega\mu - \Omega^2 \mu^2} \qquad \textbf{(S1)}$$

denotes the so-called complex susceptibility of the signaling system. Note that the phase-lag ϕ_a between $s(t)$ and $a(t)$ is independent of the base-level s_0. The oscillation amplitude $\rho_\mathrm{a} s_1$ scales as s_1/s_0, and thus, the output variable $a(t)$ displays adaptation because it responds to relative changes of the input stimulus.

Of note, the trigger variable $q(t)$ (see equation 7) oscillates only weakly in this case with an amplitude that is attenuated by a factor $|1+i\Omega\eta|^{-1}$ as compared to $a(t)$.

Exponential stimulus

We consider a stimulus baseline $s(t) = s_0 \exp(\sigma t)$ that changes in time at a rate σ that can be either positive (increasing stimulus) or negative (decreasing stimulus). In this case the output is detuned from its rest-value:

$$a(t) = 1 + \mu\sigma \,. \tag{S2}$$

The amount of detuning is set by a competition between the time-scale of adaptation (μ) and the time-scale on which the stimulus changes ($|\sigma|^{-1}$). Thus, the condition $q > \theta$ on the trigger variable q(t) is equivalent to the rate σ to exceed a threshold:

$$\sigma > \sigma^* = \frac{\theta - 1}{\mu} \,. \tag{S3}$$

The stimuli sampled by swimming cells can be approximated as a superposition of fast oscillations and a slowly changing baseline, and thus represent a superposition of these idealized cases.

A theory of steering along helical paths

The Frenet frame; curvature and torsion

The bending and twisting of a swimming path $\mathbf{r}(t)$ is characterized by its signed curvature $\kappa_p(t)$ and torsion $\tau_p(t)$. These quantities describe the dynamics of an orthonormal coordinate system that moves along $\mathbf{r}(t)$, consisting of the tangent vector $\mathbf{t} = \dot{\mathbf{r}}/v$, the normal vector $\mathbf{n} = \dot{\mathbf{t}}/|\dot{\mathbf{t}}|$, and the binormal vector $\mathbf{b} = \mathbf{t} \times \mathbf{n}$. This so-called Frenet frame rotates along the path according to the Frenet-Serret formulas:

$$\dot{\mathbf{r}} = v\mathbf{t}, \ \ \dot{\mathbf{t}} = v\kappa_p \mathbf{n}, \ \ \dot{\mathbf{n}} = -v\kappa_p \mathbf{t} + v\tau_p \mathbf{b}, \ \ \dot{\mathbf{b}} = -v\tau_p \mathbf{b} \tag{S4}$$

Note that the signed curvature is only defined up to a global choice of sign. The sign of τ_p distinguishes right-handed helices ($\tau_p > 0$) and left-handed helices ($\tau_p < 0$).

Swimming along perfect helices

Flagellar propulsion with an asymmetric, nonplanar beat pattern that is perfectly periodic in time implies an averaged swimming path $\mathbf{r}(t)$ that is a perfect helix with constant curvature κ_p and torsion τ_p. The vector $\mathbf{\Omega}(t) = v[\tau_\mathrm{p}\,\mathbf{t}(t) - \kappa_\mathrm{p}\,\mathbf{b}(t)]$ can be shown to be an invariant of this motion. In fact, $\Omega_0 = |\mathbf{\Omega}|$ is the angular frequency of helical swimming, while the helix vector $\mathbf{h} = \mathbf{\Omega}/\Omega_0$ points along the centerline of the helix. The vector $\mathbf{\Omega}$ characterizes rotations of the Frenet frame, e.g. $\dot{\mathbf{t}} = \mathbf{\Omega}\times\mathbf{t}$. Thus, the tangent vector $\mathbf{t}$ performs a precession motion around the helix vector $\mathbf{h}$ with a constant rotation rate Ω_0. We note the useful formulas $\mathbf{\Omega}\cdot\mathbf{t} = v\tau_\mathrm{p}$ and $|\mathbf{\Omega}\times\mathbf{t}| = v|\kappa_\mathrm{p}|$. The radius and pitch of the helix can be computed as $r_0 = \kappa_\mathrm{p}/(\kappa_\mathrm{p}^2+\tau_\mathrm{p}^2)$ and $2\pi h_0 = 2\pi\tau_\mathrm{p}/(\kappa_\mathrm{p}^2+\tau_\mathrm{p}^2)$, respectively. Note that planar circular paths (Fig. 2a) and twisted ribbons (Fig. 2b) can be considered as degenerate cases of helical swimming characterized by $\tau_\mathrm{p} = 0$ and $\kappa_\mathrm{p} = 0$, respectively.

Sampling a concentration gradient along helical paths

We now consider the idealized case of a cell moving along a perfect helical path $\mathbf{r}(t)$ inside a linear concentration field $c(\mathbf{x}) = c_0 + \nabla c\cdot\mathbf{x}$. Relative to the helix vector $\mathbf{h}$, the concentration gradient vector ∇c can be decomposed as

$$\nabla c = \nabla_{\|}c + \nabla_{\perp}c \qquad \textbf{(S5)}$$

with (i) a component parallel to the helix vector, $\nabla_{\|}c = (\nabla c\cdot\mathbf{h})\mathbf{h}$, and (ii) a component perpendicular to the helix vector, $\nabla_{\perp}c = \nabla c - \nabla_{\|}c$, see Fig. 3c in the main text. While swimming along a helical path, the cell samples a concentration stimulus $s(t) = c(\mathbf{r}(t))$ from the concentration field that comprises (i) a slow change of the stimulus baseline resulting from a net motion along the direction $\mathbf{h}$ and (ii) a fast oscillation with the frequency Ω_0 of helical swimming. For an appropriate choice of coordinate system, we find

$$s(t) = c_0 + (\nabla_{\|}c\cdot\mathbf{h})h_0\Omega_0 t + |\nabla_{\perp}c|\,r_0\cos(\Omega_0 t)\,. \qquad \textbf{(S6)}$$

Chemotaxis by phase-locked oscillations of path curvature and torsion

The time-dependent concentration stimulus sampled by a sperm cell along its path serves as input for a signaling system that controls the shape of the flagellar beat, and thus changes curvature $\kappa_p(t)$ and torsion $\tau_p(t)$ of the swimming path. Generally, an oscillatory concentration stimulus as in equation (S6) will elicit phase-locked oscillations of path curvature and torsion. We consider an idealized case of perfect curvature and torsion oscillations with the frequency Ω_0 of helical swimming

$$\kappa_p(t) = \kappa_0 + \kappa_1 \cos(\Omega_0 t + \phi_\kappa), \qquad \textbf{(S7)}$$

and analogously, $\tau_p(t) = \tau_0 + \tau_1 \cos(\Omega_0 t + \phi_\tau)$. Here, we explicitly account for a phase-shift ϕ_κ between oscillations of $s(t)$ and $\kappa_p(t)$, which characterizes latency times of chemotactic signal processing. Oscillating curvature and torsion yield bent helices: Using a theory put forward previously[1-3], we can compute the bending rates γ_1 and γ_2 of helical paths defined in the Methods section equation (1)

$$\gamma_1 = \frac{\Omega_0}{2}\left(r_0 \tau_1 \cos\phi_\tau - h_0 \kappa_1 \cos\phi_\kappa\right) \qquad \textbf{(S8)}$$

$$\gamma_2 = \frac{\Omega_0}{2}\left(h_0 \kappa_1 \sin\phi_\kappa - r_0 \tau_1 \sin\phi_\tau\right) \qquad \textbf{(S9)}$$

Thus, a sufficient condition for optimal alignment of the helix axis with respect to the concentration gradient characterized by $\gamma_1 > 0$ and $\gamma_2 = 0$ is given by $\phi_\kappa = \pi$ and $\phi_\tau = 0$, implying that curvature oscillations would be anti-phase to stimulus oscillations, while torsion oscillations would be in-phase. These results pertain also in more realistic cases of slightly nonlinear concentration fields, and concentration stimuli sampled along helical paths that are perturbed by steering feedback, as long as the feedback is weak (characterized by $\chi\rho_a |\nabla c| r_0 \ll 1$).

The "off response"

Whether the helical path is directed up the concentration gradient $(\nabla c \cdot \mathbf{h} > 0)$ or down the concentration gradient $(\nabla c \cdot \mathbf{h} < 0)$ is reflected by a slow increase or decrease of the stimulus baseline, respectively. From Equations (S6) and (7), we find for the trigger variable $q(t)$

$$q \approx 1 + \mu \Omega_0 \frac{\nabla c \cdot \mathbf{h}}{c} h_0 . \qquad \textbf{(S10)}$$

Thus, an *off* response is triggered in our simulations whenever the relative gradient strength in the direction of the helix axis falls below the critical value:

$$\frac{\nabla_{\|} c}{c} < \frac{\theta - 1}{\mu \Omega_0 h_0} . \qquad \textbf{(S11)}$$

For the parameters used, we have $(\theta - 1)/(\mu \Omega_0 h_0) = -1.2 \cdot 10^{-3} \mu\mathrm{m}^{-1}$ or about -6% over one helix pitch $2\pi h_0 \approx 50 \mu\mathrm{m}$. During an *off* response, the helix axis rotates rapidly as a result of large-amplitude oscillations of path curvature and torsion. While the geometric principle is fully analogous to the case of *on* responses, equations (S8) and (S9) will only hold in an approximate sense and the directional precision of these vigorous steering responses might be reduced as compared to the case of *on* responses.

Phase-lags in simulations

In our simulations, path curvature $\kappa_\mathrm{p}(t)$ and torsion $\tau_p(t)$ are regulated only indirectly by dynamically adjusting the mean flagellar curvature K_0, see equation (6). For the parameters chosen, we observe an additional phase-lag ϕ_{K_0} between oscillations of K_0 and κ_p, where $\phi_{K_0} \approx \pi/3$. This phase-lag depends on the oscillation frequency Ω and vanishes if we impose adiabatically slow oscillations of mean flagellar curvature. The effective phase-lag ϕ_κ that governs the bending rate in equation (S8) is the sum of (i) the phase-lag ϕ_a due to signaling latency and (ii) the phase-lag ϕ_{K_0} arising from the dynamic regulation of the beat pattern,

$$\phi_\kappa = \phi_a + \phi_{K_0}\,. \qquad \textbf{(S12)}$$

For the parameters chosen, we find $\phi_a \approx 2\pi/3$ according to equation (S1). Thus, $\phi_\kappa \approx \pi$. Similarly, $\phi_\tau \approx 0$. From equation (S8) and (S9), we find $\gamma_1 > 0$ and $\gamma_2 \approx 0$, corresponding to positive chemotaxis with the helix axis bending in the direction of the concentration gradient.

Location of stimulus receptor

The minimal theory presented above assumed for simplicity that stimulus concentration $s(t)$ is measured at the position $\mathbf{r}(t)$ of the sperm head, see equation (4). However, chemoattractant molecules bind to surface receptors distributed along the flagellar length [4]. We can account for this by defining a flagellar stimulus $s_w(t)$ that represents a weighted average of the local stimulus concentration along the flagellum

$$s_w(t) = \int_0^L c(\mathbf{r}_\mathbf{f}(l,t),\mathrm{t})w(l)\,\mathrm{d}l\,. \qquad \textbf{(S13)}$$

Here, $\mathbf{r}_\mathbf{f}(l,t)$ denotes the centerline of the flagellum parametrized by arc-length l and w(l) is a normalized density of receptors as a function of l. For the special case of a δ-distribution located at the head position, we recover the previous definition. We also define the trajectory of the weighted "center of mass" $\mathbf{r}_w(t)$ of the receptor ensemble

$$\mathbf{r}_w(t) = \int_0^L \mathbf{r}_\mathbf{f}(l,t)w(l)\,\mathrm{d}l \qquad \textbf{(S14)}$$

as well as the corresponding path curvature $\kappa_w(t)$ of its averaged path. We can use the stimulus $s_w(t)$ instead of the head stimulus $s(t)$ as input for the adaptation module given in equation (5). Simulation results are largely independent of the choice of $w(l)$, provided the delay time of the adaptation module is adjusted accordingly. Generally, there is a phase lag ϕ_f between the flagellar stimulus $s_w(t)$ and the head stimulus $s(t)$ such that, $s_w(t) \approx s(t + \Omega_0^{-1}\phi_f)$. This additional phase lag can be compensated by changing the delay time of the adaptation module to ensure that the effective phase lag $\phi_\kappa = \phi_a - \phi_f + \phi_{K_0}$ between oscillations of head stimulus $s(t)$ and oscillations of head path curvature $\kappa(t)$ still sum up to π. For the case of uniformly distributed receptors along the flagellum ($w(l)$ = $1/L$), we find e.g. $\phi_f \approx \pi/2$. Choosing η = 60 ms in

simulations, yielded $\phi_a \approx 5\pi/3$ and thus positive chemotaxis with $\phi_\kappa \approx \pi$. Note that equations (S8) and (S9) governing the direction of helix bending generalize to the case of an arbitrary receptor distribution $w(l)$, provided ϕ_κ is replaced by the phase lag between oscillations of flagellar stimulus $s_w(t)$ and oscillations of the weighted flagellar path curvature $\kappa_w(t)$. As anticipated, simulations accounting for a uniform receptor distribution along the flagellum do not qualitatively differ to the minimal model considered in the main text, where the stimulus concentration is measured at the position of the sperm head (see Supplementary Fig. 4).

References

1. Friedrich, B.M. Chemotaxis of Sperm Cells. PhD thesis (Technische Universität Dresden, Germany2008).
2. Friedrich, B.M. & Jülicher, F. Steering chiral swimmers along noisy helical paths. *Phys. Rev. Lett.* **103**, 068102 (2009).
3. Friedrich, B.M. & Jülicher, F. Chemotaxis of sperm cells. *Proc. Natl. Acad. Sci. USA* **104**, 13256-13261 (2007).
4. Bönigk, W. *et al.* An atypical CNG channel activated by a single cGMP molecule controls sperm chemotaxis. *Sci. Signal.* **2**, ra68 (2009).
